# Neuroprotective and neurogenic effects of novel tetramethylpyrazine derivative T-006 in Parkinson’s disease models through activating the MEF2-PGC1α and BDNF/CREB pathways

**DOI:** 10.18632/aging.103551

**Published:** 2020-07-24

**Authors:** Haiyun Chen, Jie Cao, Ling Zha, Peile Wang, Zheng Liu, Baojian Guo, Gaoxiao Zhang, Yewei Sun, Zaijun Zhang, Yuqiang Wang

**Affiliations:** 1 International Cooperative Laboratory of Traditional Chinese Medicine Modernization, Innovative Drug Development of Chinese Ministry of Education, Institute of New Drug Research, Jinan University College of Pharmacy, Guangzhou, China; 2 School of Pharmacy, Guangdong Pharmaceutical University, Guangzhou, China; 3 Foshan Stomatology Hospital, School of Stomatology and Medicine, Foshan University, Foshan, China

**Keywords:** tetramethylpyrazine derivative T-006, Parkinson’s disease, transcriptional factor myocyte enhancer factor 2D, peroxisome proliferator-activated receptor γ (PPARγ) co-activator 1α, adult neurogenesis

## Abstract

T-006, a new derivative of tetramethylpyrazine, has been recently found to protect against 6-hydroxydopamine (6-OHDA)-induced neuronal damage and clear α-synuclein (α-syn) by enhancing proteasome activity in an α-syn transgenic Parkinson’s disease (PD) model. The effect of T-006 on the 1-methyl-4-phenyl-1, 2, 3, 6-tetrahydropyridine (MPTP)-induced PD model, however, has not been tested and T-006’s neuroprotective mechanisms have not been fully elucidated. In this study, we further investigated the neuroprotective and neurogenic effects of T-006 and explored its underlying mechanism of action in both cellular and animal PD models. T-006 was able to improve locomotor behavior, increase survival of nigra dopaminergic neurons and boost striatal dopamine levels in both MPTP- and 6-OHDA-induced animals. T-006 treatment restored the altered expressions of myocyte enhancer factor 2D (MEF2D), peroxisome proliferator-activated receptor γ (PPARγ) co-activator 1α (PGC1α) and NF-E2-related factor 1/2 (Nrf1/2) via modulation of Akt/GSK3β signaling. T-006 stimulated MEF2, PGC1α and Nrf2 transcriptional activities, inducing Nrf2 nuclear localization. Interestingly, T-006 promoted endogenous adult neurogenesis toward a dopaminergic phenotype by activating brain-derived neurotrophic factor (BDNF) and cAMP responsive element-binding protein (CREB) in 6-OHDA rats. Our work demonstrated that T-006 is a potent neuroprotective and neuroregenerative agent that may have therapeutic potential in the treatment of PD.

## INTRODUCTION

Parkinson’s disease (PD) is the second most common neurodegenerative disorder and is characterized by motor and behavioral disturbances. PD is associated with progressive loss of dopaminergic (DA) neurons located in the substantia nigra pars compacta (SNc) [1–3]. Currently, anti-PD drugs used in clinic include dopamine drugs (dopamine substitution agent, dopamine receptor agonist, monoamine oxidase type b inhibitor, et al), anticholinergic drugs, etc. [4]. However, these anti-PD drugs can only improve the symptoms but cannot reverse the deterioration process of the disease. Moreover, with long-term treatment, the medication becomes less efficacious and increasingly toxic [5]. There is thus a significant unmet need for novel agents, particularly those with the disease-modifying potential to block the degeneration of SNc DA neurons.

Although the etiology of PD has not been fully elucidated, accumulating evidence suggests that mitochondrial dysfunction and oxidative stress play pivotal roles in the pathogenesis of PD. Studies in MPP^+^ and 6-OHDA-induced PD models demonstrated that mitochondrial dysfunction and oxidative stress could lead to impairment of adenosine triphosphate (ATP) production, inhibition of complex I and generation of free radicals [6, 7]. Mitochondrial dysfunction and oxidative stress can be induced by several PD associated genes [8–10], including the underexpressed peroxisome proliferator-activated receptor γ (PPARγ) co-activator 1α (PGC1α), a major regulator of mitochondrial biogenesis and energy metabolism in PD patients [11]. In contrast, activation of PGC1α resulted in upregulation of nuclear-encoded subunits of the mitochondrial respiratory chain and blocked the DA neuron loss in various neurotoxin-induced PD models [12]. However, because of PGC1α’s lack of DNA-binding activity, its function can only be realized through interaction with numerous activating transcription factors, including transcription factor myocyte enhancer factor 2 (MEF2) and nuclear respiratory factors (Nrfs) [13, 14]. Among these factors, MEF2 (A-D) plays an important role in neuronal survival in several experimental paradigms [15]. Loss of MEF2D function was associated with increased vulnerability to MPTP and 6-OHDA-induced toxicity [16, 17]. In addition, activation of MEF2D has been reported to protect DA neurons from toxin-induced death *in vitro* and *in vivo* [15, 18]. Therefore, activation of the MEF2D-PGC1α signal pathway might be a novel and effective therapeutic target for PD.

As reported, more than 50% of DA neurons in the SNc have degenerated by the time patients present with clinical symptoms [19]. It is conceivable that all neuroprotective strategies start too late, and are thus not sufficient for disease-modifying therapy. Hence, adult neurogenesis, a process of proliferation, differentiation, migration and integration of neural stem cells (NSCs) or neural progenitor cells (NPCs) *in vivo*, is being intensively studied as one of the potential modes for neurodegenerative disease therapy. There is evidence suggesting that dopamine motivates adult neurogenesis in the subventricular zone (SVZ) of the lateral ventricles (LV) as well as in the dentate gyrus (DG) of the hippocampus, where the NPCs in the brains of adult rodents and humans are mostly located [20, 21]. Decreased proliferation of NPCs in the SVZ of human PD brains and PD animal models induced by MPTP or 6-OHDA [22–24] suggests that compensatory neuron regeneration is far from enough to replace injured or dead neurons. Neuron regeneration and functional recovery via implanting differentiated neurons into the brains of PD patients has succeeded, suggesting that it is possible to replace dead neurons with regenerated neurons [25–27]; however, because of technological imperfections and ethical limitations, discovery and development of effective drugs to stimulate endogenous regeneration and restore functions of adult NPCs is still the most attractive therapeutic strategy [28].

Tetramethylpyrazine (TMP) is one of the important active ingredients in Chinese herb *Ligusticum wallichii Franch* (Chuanxiong) and has been widely used in clinic for treatment of ischemic cardiovascular and cerebrovascular diseases for decades. Previous studies have also shown that TMP has beneficial effects in PD models through multifunctional mechanisms, including free radical-scavenging, calcium channel blocking, protecting against DA neuron injury and blocking mitochondrial apoptosis [29, 30]. J147, an effective broad neuroprotectant *in vitro* and *in vivo,* is considered to have great potential to enhance memory and restore cognition in several Alzheimer’s disease (AD) models, owing to its ability to simultaneously potentiate NPCs proliferation and provide neuroprotection [31–33]. T-006 is designed by replacing the methoxyphenyl group of J147 with TMP. In our previous study, T-006 exerted multifunctional neuroprotective effects against neuronal death induced by neurotoxins and enhanced learning and memory in APP/PS1 transgenic mice [34]. Although the detailed mechanisms underlying the neuroprotective effects of T-006 have not been fully elucidated, its neuroprotection against glutamate-induced excitotoxicity has been demonstrated to be associated with inhibition of extracellular signal-regulated kinase activation and activation of phosphatidylinositol 3-kinase (PI3K)/Akt signaling [35]. Recently, pretreatment of T-006 has been found to prevent neuronal damage induced by 6-OHDA in PC12 cells and in mice [36]. In addition, T-006 promoted the clearance of α-synuclein (α-syn) by enhancing proteasome activity in the α-syn transgenic PD model [36]. However, the effect of T-006 on MPTP/MPP^+^-induced models of PD has not been tested and the neuroprotective mechanisms of T-006 not fully elucidated. It is also worth probing whether T-006 also promotes endogenous neurogenesis in a PD model, since it was recently found to increase neurogenesis in an animal model of stroke [37]. In this study, the therapeutic effects of T-006 were assessed in MPTP-induced mice and 6-OHDA-induced rats. The neurogenic effect of T-006 was tested in 6-OHDA-induced rats. Furthermore, the possible involvement of MEF2D-PGC1α signaling and BDNF/CREB signaling in neuroprotective and neurogenic effects of T-006 were explored.

## RESULTS

### Protective effect of T-006 on MPTP-induced mice

**Figure 1:**
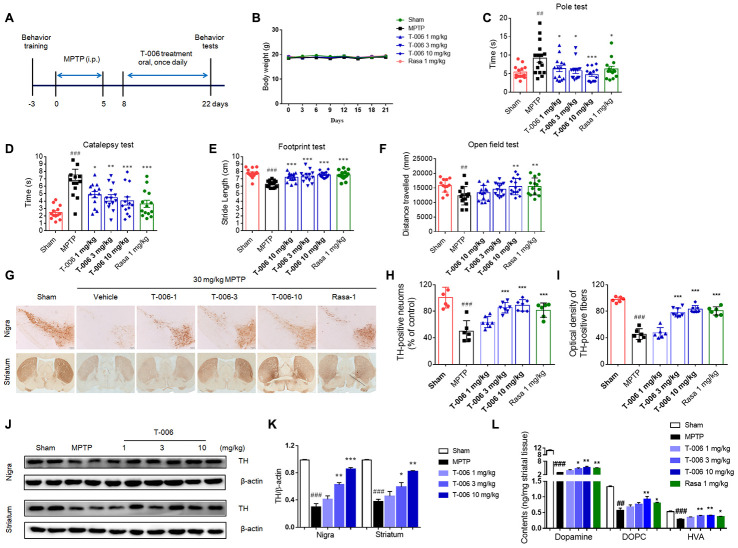
**T-006 improves motor behavior, protects nigrostriatal neurons, and suppresses disease progression in mice subjected to MPTP.** (**A**) Schedule for T-006 treatment of a chronic MPTP mice PD model. (**B**) The changes of body weight in different groups (n=14-16 per group). (**C**) Time spent on pole. (**D**) Time taken for the left forelimb to grip wire. (**E**) Distance between hind paws’ markings on white absorbing paper. (**F**) Distance travelled (cm) in open field. (**G**) Immunohistochemistry for TH in the substantia nigra (SN; upper panel) and striatum (ST; lower panel) of MPTP mice. (**H**) Stereological counting of TH-positive DA neurons from SN. (**I**) Relative density of TH-positive neuronal fibers in ST. (**J**) Representative Western blots illustrating the expression of TH in SN and ST. (**K**) Densitometric analysis of TH/β-actin of treatment with T-006 at the indicated concentrations in SN and ST. The results are shown as the mean±SEM (n=6-8 per group). (**L**) Striatal dopamine and its metabolites DOPAC and HVA were analyzed by electrochemical HPLC with 6 to 8 mice per group. Data are expressed as mean±SEM. ^#^P<0.05, ^##^P<0.01, ^###^P<0.001 vs. sham group, and ^*^P<0.05, ^**^P<0.01 and ^***^P<0.001vs. MPTP group.

We first evaluated the effect of T-006 on MPTP-induced SNc DA neuron degeneration in C57BL/6 mice, which is a widely accepted *in vivo* model of PD. The experimental procedure is illustrated in Figure 1A. During the study period, there was no significant difference in body weight among the different treatment groups (Figure 1B). Behavioral assessments of the mice showed that MPTP significantly increased climbing time in the pole test as well as the hanging time of the catalepsy test. Moreover, MPTP resulted in abnormal gait (decreased stride length) and a decrease in distance traveled during the open field test. However, treatment with T-006 and the positive control drug rasagiline significantly corrected any movement abnormalities (Figure 1C–1F). The motor disturbances are a consequence of the progressive loss of DA neurons in the SNc and a subsequent reduction in striatal dopamine levels. As expected, MPTP caused a significant loss of the TH signal in the SNc and striatum (Figure 1G–1I). Both T-006 and rasagiline treatment significantly retained the TH signal in the SNc and striatum, suggesting T-006 as well as rasagiline effectively protected DA neurons against MPTP-induced toxicity. A parallel increase in TH protein expression was observed in the SNc and striatum of the T-006 treated mice (Figure 1J and 1K). In addition, we found that MPTP induced a dramatic reduction in the striatal dopamine level as well as its metabolites DOPAC and HVA. However, T-006 and rasagiline treatment significantly attenuated the MPTP-induced decrease in both striatal dopamine and its metabolites (Figure 1L).

### Protective effect of T-006 on 6-OHDA-induced rats

**Figure 2:**
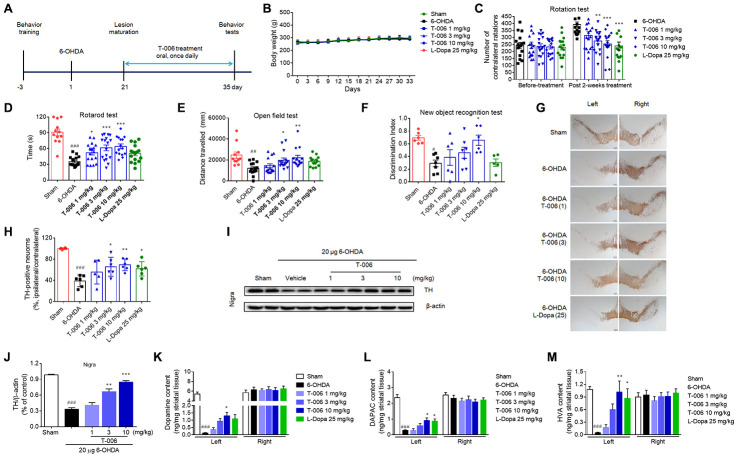
**T-006 improves motor behavior and prevents dopaminergic neurodegeneration in rats subjected to 6-OHDA.** (**A**) Schedule for T-006 treatment of a progressive 6-OHDA rat PD model. (**B**) The changes of body weight in different groups (n=12-16 per group). (**C**) Turns initiated by apomorphine-induced 6-OHDA rats. (**D**) Time spent on the rotarod. (**E**) Distance travelled (cm) in open field. (**F**) Discrimination index in new objective test. (**G**) Immunohistochemistry for TH in SN. (**H**) Stereological counting of TH-positive DA neurons from SN. (**I**) Representative Western blots illustrating the expression of TH in SN. (**J**) Densitometric analysis of TH/β-actin of treatment with T-006 at the indicated concentrations in SN. (**K**–**M**) Striatal dopamine and its metabolites DOPAC and HVA were analyzed by electrochemical HPLC with 6 to 8 mice per group. The results are shown as the mean±SEM. ^#^P<0.05, ^##^P<0.01, ^###^P<0.001 vs. sham group, and ^*^P<0.05, ^**^P<0.01 and ^***^P<0.001vs. 6-OHDA group.

Previously, we demonstrated that pretreatment with T-006 prevented neuronal damage and restored motor deficits induced by 6-OHDA in mice [36]. Here, we further validated the effect of post-lesion treatment (3 weeks after 6-OHDA injection) with T-006 on a rat model with a unilateral 6-OHDA lesion (Figure 2A). Body weight among the treatment groups exhibited no differences during the five-week experimental period (Figure 2B). Three weeks after unilateral 6-OHDA injection, the mean number of contralateral rotations induced by APO was about 200 turns in 30 min. There was no significant difference among groups before drug treatment. The rotation number of the 6-OHDA model group was markedly increased at 5 weeks after the 6-OHDA lesion when compared with the rotation number at 3 weeks (Figure 2C), suggesting that 6-OHDA caused continuous damage to the dopaminergic system. Two weeks of treatment with either T-006 or L-Dopamine (L-Dopa) significantly decreased APO-induced rotation of 6-OHDA rats in a dose-dependent manner. T-006, but not L-Dopa, treatment also reversed both the decreased retention time in the rotarod test (Figure 2D) and the decreased distance traveled during the open field test of 6-OHDA rats (Figure 2E). Interestingly, treatment with 10 mg/kg of T-006 significantly improved the impaired learning and memory function of 6-OHDA rats, as indicated by their increased discrimination index in the new object recognition test; the positive drug L-Dopa, however, did not show any effect (Figure 2F). Immunostaining results demonstrated that 6-OHDA-induced DA neuron loss in the SNc was remarkably attenuated by both T-006 and L-Dopa treatment (Figure 2G, 2H). That was further confirmed by Western blot analysis, evidenced by the higher TH protein level in the lesioned SNc of T-006-treated rats compared to that of 6-OHDA model rats (Figure 2I, 2J). In addition, injection of 6-OHDA induced a dramatic decrease in dopamine, DOPAC and HVA levels in the lesioned-side’s striatum; both T-006 and L-Dopa significantly increased the levels of dopamine, DOPAC and HVA compared with those of 6-OHDA rats (Figure 2K–2M).

### Regulation of T-006 on MEF2D-PGC1α and Akt/GSK3β signaling pathway in *in vivo* PD models

**Figure 3:**
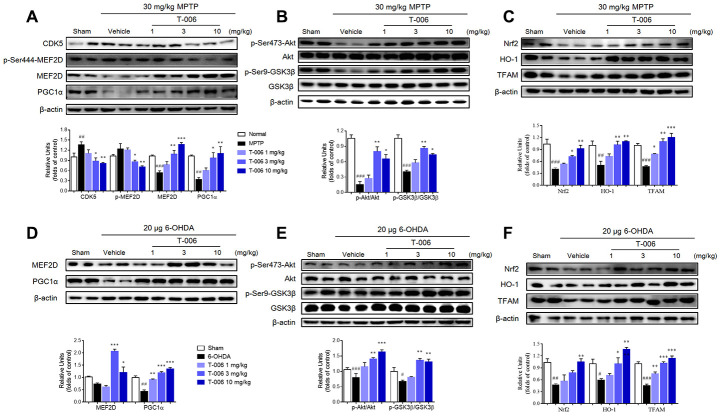
**T-006 stimulates MEF2D/PGC1α/Nrf2 signal pathway through regulation of the Akt/GSK3β pathway in PD animal models.** (**A**) Representative Western blots and densitometric analysis the expression of CDK5, p-MEF2D, MEF2D and PGC1α. (**B**, **E**) Representative Western blots and densitometric analysis of the expression of p-Akt and p-GSK3β. (**C**, **F**) Representative Western blots and densitometric analysis of the expression of Nrf2, HO-1 and TFAM. (**D**) Representative Western blots and densitometric analysis the expression of MEF2D and PGC1α. Data are expressed as mean±SEM (n=3 per group). ^#^P<0.05, ^##^P<0.01, ^###^P<0.001 vs. sham group, and ^*^P<0.05, ^**^P<0.01 and ^***^P<0.001vs. MPTP or 6-OHDA group.

**Figure 4:**
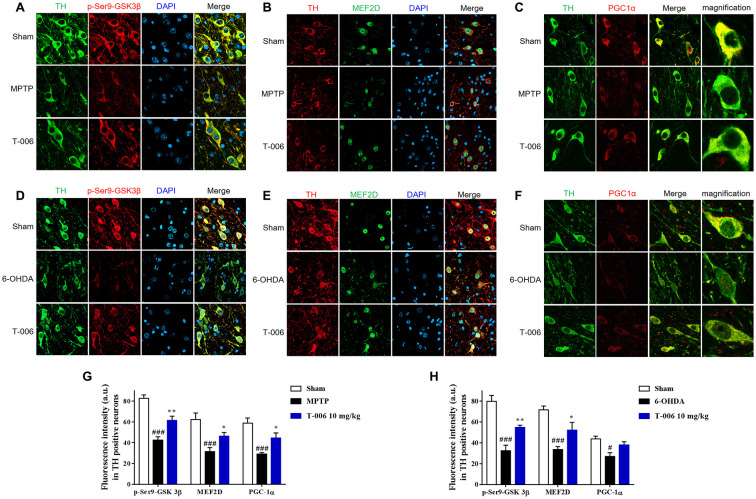
**T-006 prevents the** loss of **SN DA neuron loss by activating MEF2D/PGC1α/GSK3β signal pathway.** Representative images of middle brain sections co-stained with antibodies against (**A**, **D**) TH (green) and p-GSK3β (red); (**B**, **E**) TH (red) and MEF2D (green); (**C**, **F**) TH (green) and PGC1α (red). DAPI (blue) indicates nucleus. (**G**, **H**) Quantitative analysis of immunofluorescence intensity in TH-positive cells. Data are expressed as mean±SEM (n=3 to 4 per group). ^#^P<0.05, ^###^P<0.001 vs. sham group, and ^*^P<0.05, ^**^P<0.01 vs. MPP^+^ or 6-OHDA group.

Increasing evidence indicates that MEF2, especially MEF2D in particular, plays a critical role in the survival of DA neurons [18, 38, 39], and previous work reported that activation of MEF2D transcription effected potent protection against MPP^+^-induced neuronal damage [40]. Therefore, the possibility that T-006 might activated the MEF2D-PGC1α signaling pathway to protect DA neurons in PD models was examined here. As shown in Figure 3A and 3D, both MPTP and 6-OHDA decreased MEF2D and PGC1α proteins’ expression in the SNc of PD models. Meanwhile, MPTP and 6-OHDA modulated the upstream and downstream molecules of MEF2D and PGC1α. It has been reported that CDK5 and GSK3β serve as negative regulators of MEF2D in response to diverse toxic signals relevant to PD [41]. MPTP increased CDK5 protein expression (Figure 3A), which promoted the phosphorylation of MEF2D at Ser444 to destabilize MEF2D. Both MPTP and 6-OHDA decreased p-Ser473-Akt and p-Ser9-GSK3β expression (Figure 3B, 3E). Nrf2, HO-1 and TFAM, are downstream molecules transcriptionally activated by MEF2D and PGC1α; all were dramatically decreased by both MPTP and 6-OHDA induction (Figure 3C, 3F). All of above altered protein expressions were obviously restored by T-006 treatment (Figure 3). In addition, the effect of T-006 on expression of MEF2D, PGC1α and p-GSK3β in DA neurons was further confirmed by double immunofluorescence staining of brain SNc sections. As shown in Figure 4, MPTP and 6-OHDA obviously decreased the density of TH-positive neurons and reduced p-Ser9-GSK3β, MEF2D and PGC1α fluorescent signals in TH-positive neurons. T-006 treatment clearly increased the density of TH-positive neurons, and restored the p-Ser9-GSK3β, MEF2D and PGC1α fluorescent signals in them.

### Protective effects of T-006 on MPP^+^- and 6-OHDA-induced CGNs injury

**Figure 5:**
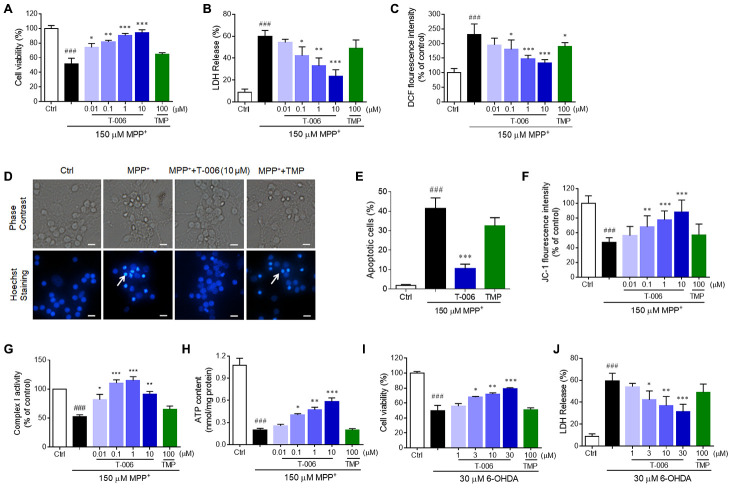
**T-006 protects against neurotoxin-induced neurotoxicity in neurons.** CGNs or corrected DA neurons were pretreated with T-006 at the indicated concentration for 2 hours followed by 150 μM MPP^+^ or 30 μM 6-OHDA treatment for 24 hours. (**A**, **I**) MTT assays to evaluate the cell viability. (**B**, **J**) Measurements of the LDH release. (**C**) ROS production assessed by DCF fluorescence intensity. (**D**) Hoechst staining to show the apoptotic cells. (**E**) Statistical analysis of the number of pykonitic nuclei of (**D**). (**F**) Mitochondrial membrane potential tested by JC-1 kit. (**G**) Complex I activity evaluation. (**H**) Intracellular ATP content measurement. Results are representative of three independent experiments as the mean±SEM. ^###^P<0.001 vs. control (Ctrl) group, and^*^P<0.01, ^**^P<0.01 and ^***^P<0.001 vs. MPP^+^ or 6-OHDA group.

CGNs are the most abundant and homogeneous neuronal types in the mammalian central neural system, which have been widely used as an *in vitro* model for the evaluation of candidate neuroprotectants [42–45]. Thus, the protective effects of T-006 against MPP^+^- and 6-OHDA-induced neurotoxicity were further examined in primary CGNs. T-006 pretreatment significantly increased cell viability while decreasing LDH release and intracellular ROS over-production in MPP^+^-treated CGNs, all in a concentration-dependent manner (Figure 5A–5C). T-006 reduced neuronal apoptosis induced by MPP^+^ through inhibiting the decrease in mitochondrial membrane potential (Figure 5D–5F). MPP^+^ is a selective inhibitor of mitochondrial complex I. Thus the impact of T-006 on mitochondrial complex I activity and ATP content was evaluated. As expected, T-006 significantly reversed the decrease in complex I activity induced by MPP^+^ and increased ATP content in CGNs (Figure 5G, 5H).

Next, we evaluated the protective effect of T-006 on 6-OHDA-induced CGN injury. Similarly, T-006 pretreatment remarkably increased cell viability and decreased LDH release in a concentration-dependent manner in 6-OHDA-induced CGNs (Figure 5I, 5J). The minimal effective concentration of T-006 on MPP^+^- and 6-OHDA-induced models was 0.1 and 3 μM, respectively. However, TMP, the parent compound of T-006, exhibited no significant protective effect against MPP^+^- and 6-OHDA-induced neurotoxicity at the tested concentration of 100 μM (Figure 5).

### Involvement of MEF2, PGC1α and Nrf2 activation in the protection of T-006 against neurotoxicity *in vitro*

**Figure 6:**
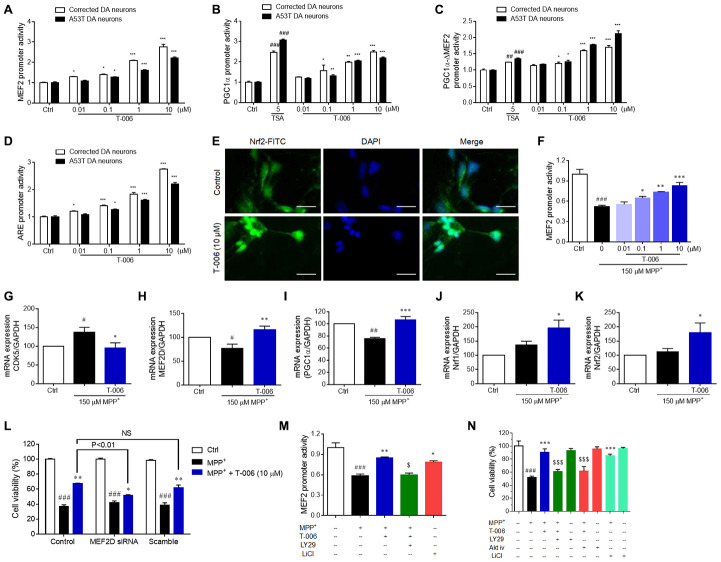
**T-006 activates the MEF2/PGC1α/Nrf2 pathway through regulation of the Akt-GSK3β pathway.** A53T and corrected DA neurons treated with T-006 and the positive drug at the indicated concentration for 24 hours. For MPP^+^-treatment assay, CGNs were pretreated with or without LY294002 (1 μM), Akt-iv (1 μM), or LiCl (10 μM) for 2 h, incubated with or without T-006 for 2 h, and finally exposed to MPP^+^. Cell viability was examined using an MTT assay. Luciferase reporter gene assays respectively included MEF2 (**A**), PGC1α (**B**), PGC1α-ΔMEF2 (**C**) and ARE (**D**). (**E**) Representative images of neurons co-stained with antibody against Nrf2 (green). DAPI (blue) indicates nucleus. (**F**) Luciferase reporter gene assays of MEF2 with MPP^+^ induction. (**G**–**K**) respectively represent the fold changes of CDK5, MEF2D, PGC1α, Nrf1 and Nrf2 at mRNA level. (**L**) Effect of MEF2D reduction on MPP^+^-induced neurotoxicity in CGNs. (**M**) Effects of Akt pathway inhibitor LY294002 and GSK3β inhibitor on MEF2 transcriptional activity. (**N**) Effects of Akt pathway inhibitors LY294002 and Akt-iv, and GSK3β inhibitor on MPP^+^-induced neurotoxicity in CGNs. Data above are all from three independent experiments, expressed as mean±SEM. ^#^P<0.05, ^##^P<0.01 and ^###^P<0.001 vs. control (Ctrl) group, and ^*^P<0.05, ^**^P<0.01, ^***^P<0.001 vs. MPP^+^ group.

To further investigate the potential involvement of MEF2, PGC1α and Nrf2 activation in T-006’s protective effect against neurotoxicity *in vitro*, the luciferase reporter assay was applied in A9 DA neurons derived from A53T DA neurons and isogenic-corrected ones. A53T DA neurons or corrected DA neurons were transfected with plasmas containing a MEF2 promoter, PGC1α promoter, PGC1α-ΔMEF2 (PGC1a promoter lacking the MEF2-binding site), or antioxidant response element (ARE). They were then treated with T-006 for 24 h. As shown in Figure 6A–6D, T-006 significantly increased the transcriptional activities of MEF2, PGC1α and Nrf2 in a concentration-dependent manner both in corrected DA neurons and in A53T DA neurons. The minimal effective concentration was 0.1 μM, the same as the *in vitro* neuroprotective concentration. Importantly, T-006 still enhanced the PGC1α-ΔMEF2 activity without a MEF2-binding site, suggesting T-006 can activate PGC1α independent of MEF2. Trichostatin A (TSA), a histone deacetylase inhibitor used as a positive control, also increased both PGC1α and PGC1α-ΔMEF2 promoter activity. TSA has been reported to activate PGC1α through changed chromatin assembly at the PGC1α promoter [46]. Nrf2 nuclear translocation stimulated by T-006 was further investigated in corrected DA neurons. T-006 pretreatment obviously increased Nrf2 accumulation in the nucleus (Figure 6E). Consistent with previous findings [15], MEF2 transcriptional activity was dramatically inhibited by MPP^+^ exposure, and this inhibition was significantly attenuated by T-006 pretreatment (Figure 6F).

In addition, we examined the impact of T-006 on MEF2D, PGC1α, and Nrf2 mRNA levels in CGNs exposed to MPP^+^. Similar to the protein expression results in rodent models, T-006 significantly restored the altered mRNA expression of CDK5, MFF2D, and PGC1α (Figure 6G–6I). MPP^+^ did not largely impact mRNA expression of Nrf1 and Nrf2, while T-006 significantly up-regulated Nrf1 and Nrf2 mRNA levels in the presence of MPP^+^ (Figure 6J, 6K). More importantly, the genetic silence of MEF2D by specific siRNA significantly abolished the neuroprotective effects of T-006 against MPP^+^-induced CGNs death, suggesting that the neuroprotective effect of T-006 was mainly through activation of MEF2D (Figure 6L). To confirm whether T-006 activated the MEF2 activity through regulating the Akt/GSK3β pathway, the impact of LY294002 (a specific inhibitor of PI3K) on MEF2 activation by T-006 was evaluated in MPP^+^-induced CGNs. The results showed that LY294002 completely abolished the MEF2 transcriptional activation exerted by T-006. At the same time, LiCl, a specific inhibitor of GSK3β, notably activated MEF2 transcriptional activity (Figure 6M). Consequently, LY294002 and an Akt inhibitor Akt-iv significantly attenuated the neuroprotection exerted by T-006. Also, LiCl treatment alone substantially protected cells (Figure 6N). Therefore, we concluded that T-006 protected CGNs against MPP^+^-induced neurotoxicity partly through activating MEF2D via regulation of the Akt/GSK3β pathway.

### Regenerative role of T-006 in damaged DA neurons of 6-OHDA-treated rats

**Figure 7:**
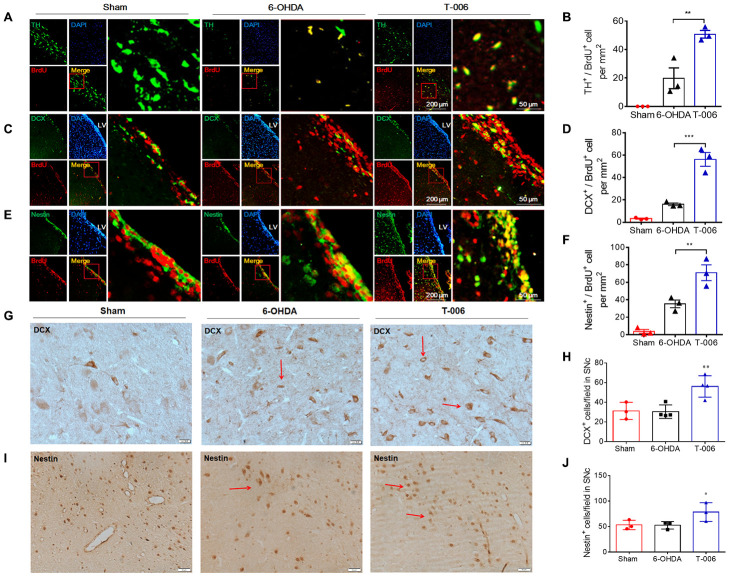
**T-006 enhances neural reconstruction by stimulating neurogenesis in rat model of PD.** Immunofluorescence images of ipsilateral hemisphere sections co-stained with antibodies against (**A**) TH (green, a marker of DA neurons) and BrdU (red, a marker of proliferating cells); (**C**) DCX (green, a marker of migrating neuroblasts) and BrdU (red); (**E**) Nestin (green, a marker of NPCs) and BrdU (red) cells. DAPI (blue) indicates nucleus. Insets show a higher magnification view of double-positive cells. Scale bar = 200 μm for whole slice, 50 μm for inset magnification. (**B**, **D** and **F**) Quantitative analysis of newly formed mature neurons (BrdU^+^/TH^+^, **D**), in the SN, migrating neuroblasts (BrdU^+^/DCX^+^, **E**) and proliferating NPCs (BrdU^+^/Nestin^+^, **F**) in the SVZ. (**G** and **I**) Immunohistochemical images of ipsilateral hemisphere sections co-stained with antibodies against DCX and Nestin in the SNc. (**H** and **J**) Quantitative analysis of DCX and Nestin-positive cells in SNc. Data are expressed as mean±SEM (n=3 to 4 per group). ^*^P<0.05, ^**^P<0.01 and ^***^P<0.001 vs. 6-OHDA group.

Our recent study demonstrated that T-006 exhibited a neurogenic effect in a rat model of ischemic stroke [37]. This study thus examined whether T-006 also has a neurogenic effect in 6-OHDA-treated rats. In the sham group, almost no TH^+^/BrdU^+^ cells were found in the SNc. However, the number of TH^+^/BrdU^+^ cells was obviously increased in the SNc of 6-OHDA-treated PD rats. Importantly, the number of TH^+^/BrdU^+^ positive cells in T-006-treated rats were much higher than that in 6-OHDA model rats, suggesting T-006 elicited an increase in the generation of new DA neurons in the injured SNc (Figure 7A, 7B). In the LV region of PD rats, the number of DCX^+^/BrdU^+^ neuroblasts and Nestin^+^/BrdU^+^ NPCs was markedly increased compared with the sham groups. Furthermore, the number of DCX^+^/BrdU^+^ neuroblasts and Nestin^+^/BrdU^+^ NPC cells in the T-006-treated group was significantly higher than that in 6-OHDA model rats (Figure 7C–7F), suggesting that newborn DA neurons found in the SNc possibly originated from the precursors existing in the SVZ of LV region. In accordance with the increase in DCX^+^ and Nestin^+^ positive cells in the LV region by T-006 treatment, a parallel increase in DCX^+^ and Nestin^+^-stained cells in the SNc area was observed (Figure 7G–7J). These results suggested that T-006 promoted DA neurogenesis through stimulating the proliferation of new progenitors in the SVZ as well as in the SNc of the adult rats.

**Figure 8:**
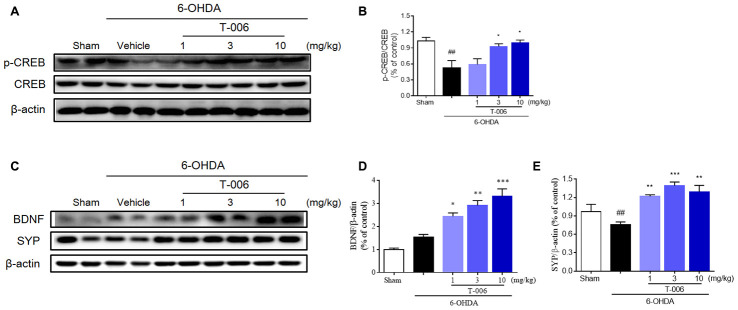
**T-006 activates BDNF/CREB signaling pathway to provide neuro-repair in PD rats.** Representative immunoblotting (**A**, **C**) and quantification of the relative protein level of p-CREB/CREB (**B**), BDNF (**D**) and synaptophysin (SYP, **E**) in the brain infarct region of sham, vehicle or T-006-treatment rats in 6-OHDA-induced PD rats. Data are expressed as mean±SEM (n=3 to 4 per group). ^##^P< 0.01 vs. sham group, and ^*^P<0.05, ^**^P<0.01, ^***^P<0.001 vs. 6-OHDA group.

Several key signaling pathways, including BDNF/TrkB and cAMP response element-binding protein (CREB), may be essential for neurogenesis from NSCs, as well as for subsequent migration and maturation [47, 48]. Therefore, we explored the involvement of these molecular events in the mechanism underlying the neurogenic effect of T-006. As shown in Figure 8A and 8B, 6-OHDA dramatically decreased the expression of p-CREB in the SNc while 3 and 10 mg/kg of T-006 treatment almost completely reversed the down-regulation of p-CREB by 6-OHDA. In accordance with the p-CREB results, T-006 treatment also significantly increased the expression of BDNF when compared with the 6-OHDA model group (Figure 8C, 8D). Synaptophysin (SYN), one of the major synaptic proteins, was found profoundly decreased in the 6-OHDA-treated rats, while T-006 treatment significantly enhanced the expression of SYN (Figure 8C, 8D), implying that T-006 possibly induced formation of functional synaptic connectivity between host neurons and NPC-derived new neurons.

## DISCUSSION

In clinic, most drugs for PD treatment only offer symptom relief with limited effect on disease progression. Similar to other neurodegenerative diseases, more than 50% of the DA neurons of the SNc are already degenerated by the time PD is diagnosed, which makes the development of efficacious neuroprotective therapy even more challenging. An alternative approach to maintaining a functional number of DA neurons is to identify compounds that stimulate the proliferation of endogenous NSCs with which to replace any lost neurons. However, unless a neuroprotective drug is also neurogenic, the prevention of neuronal loss will not be sufficient to stop disease progression. In the present study, we discovered that T-006, a novel derivative of TMP, protected SNc DA neurons against neurotoxicity induced by MPTP and 6-OHDA, and improved their motor function impairment in rodent models. For the first time, we demonstrated that T-006 could promote endogenous neurogenesis to repair the neuronal damage induced by 6-OHDA. The combined properties of neuroprotection and neurogenesis in a single molecule are likely to be more effective for the treatment of PD than any individual activity alone.

Our recent study shows that pretreatment with T-006 (2 days before 6-OHDA lesion) prevents SNc DA neuron loss and restores motor functions in the 6-OHDA-induced mice model [36]. However, T-006’s therapeutic effect when used as a post-injury treatment is more relevant to determining its potential efficacy in a clinical setting rather than simply measuring a pre-injury treatment effect. In our present study, T-006 treatment was initiated at 8 days after MPTP and 3 weeks after 6-OHDA injection, by which time DA neurons had been damaged and PD models were maturely established [49, 50]. T-006 was also able to improve locomotor behavior, increase survival of SNc DA neurons, and boost striatal dopamine levels. More importantly, in the 6-OHDA rat model, the mean number of APO-induced rotations at 3 weeks post-lesion was about 200 turns within 30 min, increasing up to 400 turns within 30 min at 5 weeks post-lesion; this suggests that 6-OHDA caused continuous damage to the dopaminergic system. Two weeks of T-006 post-lesion treatment, however, significantly attenuated the APO-induced rotation of 6-OHDA rats and increased TH expression in the lesioned side of the SNc. Although it is hard to judge whether T-006 halted or slowed the continuous damage experienced by DA neurons (neuroprotection) or whether it instead regenerated new DA neurons (neurorestoration/neuroregeneration) in 6-OHDA rats, T-006 most likely had delayed the progression of PD.

There has been increasing recognition that a substantial proportion of PD patients show concomitant cognitive impairments [51]. The incidence of dementia in PD patients is 6-12 times higher than in age-matched controls [52]. Encouragingly, treatment with T-006, but not L-Dopa, significantly improved the impaired learning and memory function of 6-OHDA rats. This is consistent with the previous finding that T-006, as well as the related compound J147, enhanced learning and memory in APP/PS1 transgenic mice [32, 34].

PGC1α is a transcriptional co-activator that controls the transcription of many genes involved in cellular metabolism including mitochondrial biogenesis, respiration and ROS metabolism [53]. Researchers found that low expression of PGC1α targeting genes existed in SNc DA neurons in earlier stages of PD [12]. Enhancing PGC1α expression or using small molecule PGC1α activators can increase mitochondrial biosynthesis in PD models and protect DA neurons from injury [54, 55]. Both our previous and present studies showed that T-006 can up-regulate PGC1α and mitochondrial biosynthesis-related molecules, including Nrf1/2 and TFAM, and thereby exerts a neuroprotective effect *in vitro* and *in vivo*. Recently, a protein kinase A (PKA)/CREB-dependent regulation has been characterized in activation of PGC1α by T-006 [36]. Apart from PKA/CREB, a large number of signaling pathways have been proposed to activate PGC1α [56]. Ryan A et al. used bioinformatics analysis and ChIP-qPCR validation to identify PGC1α as being transcriptionally stimulated by MEF2; dysfunctional MEF2 might elicit mitochondrial damage and neuronal death [57]. Prior work had also suggested that PGC1α and MEF2 were functionally linked [13, 58]. In the present study, although T-006 significantly increased MEF2D protein and mRNA expression as well as transcriptional activity, the result of the luciferase reporter assay revealed that T-006 can activate PGC1α in both MEF2 dependent and independent manners.

Pathways including p38 MAPK, PKA, CDK5 and Akt-GSK3β all phosphorylate MEF2D directly to control its activity [41]. We found that T-006 down-regulated CDK5 expression, and up-regulated p-Akt and p-GSK3β expression in both the MPTP and 6-OHDA models. It has been reported that CDK5 and GSK3β serve as negative regulators of MEF2D in response to the diverse toxic signals relevant to PD [41]. Increased CDK5 expression promoted the phosphorylation of MEF2D at Ser444, which led to destabilization of MEF2D and its activity [59]. Akt activation increased phosphorylation of GSK3β at Ser9, which inhibited GSK3β activity. Low levels of nuclear GSK3β activity were correlated with an increase in MEF2 DNA binding and transcriptional activities. In our study, treatment with LiCl, a well-known inhibitor of GSK3β, alone could promote MEF2 promoter activity and protect against MPP^+^-induced neurotoxicity. LY29004, a specific inhibitor of PI3K, significantly attenuated MEF2 promoter activity stimulated by T-006. Accordingly, MEF2D siRNA, LY29004 and Akt inhibitor (Akt-iv) all notably abolished the neuroprotective effect of T-006. These results suggest that T-006 activates MEF2D through the Akt/GSK3β pathway.

Currently, it is widely accepted that neurogenesis persists beyond embryonic neurogenesis in humans in the SVZ of the LV and DG of the hippocampus. The generation of new neurons in these regions may contribute to endogenous repair mechanisms after brain damage and chronic disease [60]. However, a deficiency of neurogenesis occurs in the SVZ and DG of human PD brains and animal models of PD [23, 24]. Hence, promoting endogenous neurogenesis with new therapeutic agents holds great promise in helping to replace the DA neurons lost in PD. Small molecules with a strong impact upon adult neurogenesis have been identified by pharmacological screens, but few of them have been tested in PD models [61]. TMP, the parent compound of T-006, has been reported to both promote proliferation and differentiation of NPCs [62, 63] as well as enhance migration toward the ischemic region in a rat model of middle cerebral artery occlusion (MCAo) [64]. J147 stimulated the *in vivo* proliferation of NPCs in the DG and SVZ of old mice [33]. Our recent study demonstrated that T-006 exhibited a neurogenic effect in a rat model of MCAo [37]. Our *in vivo* results demonstrated that, when treated with T-006, a larger population of newborn DA neurons (TH^+^/BrdU^+^ cells) came to be in the SNc; furthermore, NPC (Nestin^+^/BrdU^+^ cells) proliferation in the LV region of 6-OHDA rats was enhanced. Our results also showed an increase, induced by T-006 treatment, in the number of DCX^+^/BrdU^+^ cells (dividing and migrating neuroblasts) in the LV region of lesioned rats; this suggests that the newly generated neurons found in the SNc after T-006 treatment might originate from the SVZ of the LV region. Although some of the newborn DA neurons generated in the SNc can be derived from the SVZ, other neurogenic regions cannot be ignored. In the present study, we observed that increased Nestin^+^ and DCX^+^ cells were present in the SNc after T-006 treatment. Similar results have been previously found by others; the presence of BrdU positive DA neurons in the SNc has been hypothesized to have originated from precursor cells migrating either from the SVZ of the LV or from precursor cells already existing in the SNc [65].

The mechanism of action underlying the neurogenic effect of T-006 seems to be mediated by BDNF stimulation and the subsequent activation of the transcriptional factor CREB by phosphorylation. These results are in agreement with previous studies that show that the BDNF-CREB pathway plays an important role in adult neurogenesis [37, 65, 66]. Our present study suggests that T-006 promoted the generation of newborn neurons with a DA phenotype in 6-OHDA rats, possibly through activation of the BDNF-CREB pathway. To investigate the potential direct cellular protein targets of T-006, we have preliminarily identified that T-006 acts on the mitochondrial-related protein alpha-F1-ATP synthase (ATP5A) by the assay of drug affinity responsive target stability (DARTS) (data not shown).

Taken together, T-006 not only exerts significant, multifunctional neuroprotection upon surviving DA neurons, but it also induces endogenous neurogenesis to replace damaged cells. Together with our recent finding that T-006 exerts a protective effect against α-syn-induced neurotoxicity in A53T-α-syn transgenic mice by promoting the degradation of α-syn [36], our results suggest that T-006 holds great promise as therapeutic new strategy for treatment of PD.

## MATERIALS AND METHODS

### Materials

All media and supplements used for cell cultures were purchased from Invitrogen (Carlsbad, CA, USA). The cytotoxicity detection kit was obtained from Roche (Indianapolis, IN, USA). The Dual-Glo luciferase assay kit was obtained from Promega (Madison, WI, USA). MPTP, MPP^+^, 6-OHDA and Hoechst 33342 were purchased from Sigma-Aldrich (St. Louis, MO, USA). LY294002 and GSK3 inhibitor II were purchased from EMD Calbiochem (San Diego, CA, USA). Antibodies against phospho-Ser-473 Akt, phospho-Ser-9 GSK3, Akt, GSK3 and β-actin were acquired from Cell Signaling Technology (Danvers, MA, USA). Antibodies against CDK5, PGC1α, hemeoxygenase-1 (HO-1), Nrf2 and mitochondrial transcription factor A (TFAM) were acquired from Santa Cruz Biotechnology (Santa Cruz, CA, USA). Unless otherwise noted, all other reagents were obtained from Sigma-Aldrich.

### Ethics statement

All animal protocols were conducted under the experimental Animal Care and Use Committee of Guangzhou University of Chinese Medicine guidelines. The animal researches were approved by the Ethics Committee on Animal Experiments of Guangzhou University of Chinese Medicine.

### MPTP mouse model of PD

Forty-eight male and 38 female C57BL/6 mice (10-12 weeks old, 25±2 g) were purchased from Laboratory Animal Center of Guangdong Province. All above mice were housed in the isolated cages of the Center for Laboratory Animals at Guangzhou University of Chinese Medicine on a temperature kept at 22-24 ^o^C and 12 h light/dark cycle. Food and water were available with a standard diet ad libitum.

The sub-acute MPTP schedule was described as previously reported [67]. Mice were divided into two big groups by gender successively. Excluding the sham group, the mice were injected intraperitoneally with MPTP (Sigama-Aldrich) at a dose of 30 mg/kg once per day for five consecutive days.

Mice in the control group were received equivalent volumes of 0.9% saline (n=14, 8 males and 6 females). Three days after the last MPTP injection, mice were randomly separated into five groups and administrated intragastrically with either T-006 dissolved in olive oil at a dose of 1 mg/kg (n=14, 8 males and 6 females), 3 mg/kg (n=14, 8 males and 6 females) or 10 mg/kg (n=14, 8 males and 6 females), or with positive drug rasagiline dissolved in 0.9% saline at a dose of 1 mg/kg (n=14, 8 males and 6 females), once per day for 2 weeks. The remaining mice that had received MPTP injections (i.e. the MPTP-induced PD model group: n=16, 8 males and 8 females) as well as the sham group were treated with equivalent volumes of olive oil. At the end of drug administration, behavioral assessments were conducted.

### 6-OHDA rat model of PD

A total of 46 male and 46 female Sprague Dawley (SD) rats (Laboratory Animal Center of Guangdong Province) with 250±20 g body weight were used for the unilateral 6-OHDA-lesioned model. The rats were fed under the environmental conditions of ambient temperature 22-24 ^o^C with 45-65% humidity and 12 h light cycle and provided with adequate food and water. Animals were anesthetized with 2.5-3% isoflurane and fixed on a stereotactic frame. A craniotomy was performed at coordinates relative to the bregma and dura. All procedures were conducted under the rat brain atlas guideline from Paxinos and Watson [68]. Using a Hamilton micro-syringe, two coordinate points of the left striatum (AP 0, ML 3, DV -5.5 and -4.5) of the rat were injected into 2 μL 6-OHDA (5 mg/mL) at a rate of 1 μL/min. with rats in the sham group receiving equivalent volumes of 0.9% saline instead (n=12, 6 males and 6 females). The wound was closed using re-absorbable sutures and penicillin injected to prevent infection. Twenty-one days later, rats injected with 6-OHDA were intraperitoneally injected with 0.5 mg/kg apomorphine to induce the rats to exhibit a rotating motion to their contralateral sides; their rotary motion was recorded 10 min later for a total of 30 min. The numbers of rats that rotated between 100 and 450 turns in 30 min were selected as successful 6-OHDA-induced PD models for this study. All rats were randomly divided into five groups and administrated intragastrically with either T-006 dissolved in olive oil at a dose of 1 mg/kg (n=16, 8 males and 8 females), 3 mg/kg (n=16, 8 males and 8 females) or 10 mg/kg (n = 16, 8 males and 8 females), or with positive drug L-dopamine dissolved in olive oil at a dose of 25 mg/kg (n=16, 8 males and 8 females), once per day for 2 weeks. The remaining successfully 6-OHDA-induced rats (i.e. the 6-OHDA-induced PD model group: n=16, 8 males and 8 females) as well as the sham group were treated with equivalent volumes of olive oil. At the end of drug administration, behavioral assessments were conducted.

For the neurogenesis study, an additional 24 male rats were screened and randomly placed into three groups: sham (n=8), 6-OHDA (n=8) and T-006 treatment (10 mg/kg, n=8). The procedure was the same as the above 6-OHDA-induced PD assay, with the only difference being that twenty-eight days after 6-OHDA administration, rats were intraperitoneally injected with 50 mg/kg 5’-bromo-2’-deoxyuridine (BrdU) twice a day for five days. After 5-day-injection, all animals were sacrificed for the identification of proliferating cells.

### Behavior tests

To measure motor coordination and spontaneous locomotion, the pole-climbing, catalepsy, footprint and open field tests were executed at day 17 after last MPTP injection. Meanwhile, except the new objective recognition test only carried out in female mice, the rotation, rotator as well as open field tests were measured in both male and female 6-OHDA-induced rat PD model. All behavioral experiments were double-blinded and repeated three times to take the average for statistics analyses.

### Pole test

The method was followed the protocol initially described by Ogawa et al. [69]. The pole test consisted of a 50 cm high iron pole, 0.5 cm in diameter, wrapped in gauze to prevent slipping, with the base positioned in the home cage. The climbing rod test device is an iron pole (50 cm high, 0.5 cm in diameter) whose base is placed in a cage and the surface is wrapped with gauze. The time of at which the mice turned around to face nose downward (inversion time) as well as the total time required to climb from top to bottom were recorded.

For the catalepsy and footprint tests, we followed the procedures described by Richter et al. with little modification [70].

### Catalepsy test

The catalepsy response was measured by calculating the time (in seconds) it took for mice to use their left forelimbs to grip the wire in a block test. The mice were first placed with their right forelimbs on a block (4 cm high); the time that the animals were able to maintain this position before their left forelimbs gripped the wire was noted.

### Footprint test

Mice were trained to walk through a wooden trough (5-cm-wide, 85-cm-long) which hind paws were colored with black ink. Their footsteps were recorded on white paper and the mice’s stride length recorded.

### Open field test

The open-field test was carried out for evaluating the mice and rats’ ambulatory behavior. 10-min test sessions were recorded as described previously [71]. Animals were placed individually in an acrylic apparatus (50 cm × 50 cm × 40 cm) with a floor divided into 25 × 25 cm equal squares, then left to freely explore the arena. Observer positions and tasks did not vary during the study. The arena was cleaned with 70% ethanol solution and dried after testing each mouse to avoid the presence of olfactory cues.

### Apomorphine (APO)-induced rotation test

Rotational behavior was initiated by apomorphine (5 mg/kg) three weeks after 6-OHDA treatment and measured by an automated Rotacounter system (Zhenghua Biological Instrument Equipment Co., Ltd., China). Three weeks after receiving intrastriatal 6-OHDA injections, rats that exhibited at least 100 contralateral rotations over 30 minutes were deemed suitable for future study after receiving APO (0.5 mg/kg, i.p.).

### Rotarod test

The rotarod protocol was modified from a previous method [72]. Briefly, the Rotamex system (Zhenghua Biological Instrument Equipment Co., Ltd., China) was set to accelerate from 20 to 60 rpm with speed increases of 10 rpm/5 seconds. The rotating rod automatically stopped at 120 seconds. The spent time of animals’ movement on the rod were recorded (a maximum of 2 min was recorded).

The quantitative analyses for the above behavior tests were performed following a minimum of three independent trials by observers blinded to the compounds administration.

### Immunohistochemistry and immunofluorescence

For immunohistochemical analysis, animals were anesthetized with pentobarbital sodium (50 mg/kg; i.v.) after the behavioral experiment, and subsequently were perfused through the heart with PBS, then fixed with 4% paraformaldehyde. The striatum and substantia nigra were embedded in paraffin, which were sectioned in 5-μm-thick serial coronal sections for immunohistochemical and immunofluorescence staining. The immunohistochemical staining images that exhibited tyrosine hydroxylase (TH, 1:1000, Sigma Aldrich) positive, DCX (1:500, Cell Signaling Technology) positive, Nestin (1:500, Novus) positive and Nissl (Beyotime) positive cells in the SN areas were identified with a fluorescence microscope (Olympus, Japan). Double immunofluorescent images that exhibited TH (Merck)/MEF2D (BD Biosciences), TH/PGC1α (Novus, USA), BrdU (Cell Signaling Technology)/TH and TH/phospho-Ser9-GSK3β (p-GSK3β) cells in the SNc area and BrdU/Nestin and BrdU/DCX cells in the SVZ area were identified with a confocal fluorescence microscope (Leica, Germany). Three randomly selected, well-defined high magnification (400×) fields per animal were taken to counted positive cells, of which was randomly collected brain tissue for staining from 3 or 4 rats per group. Results were expressed as the average numbers of positive cells in unit area per section of six to eight animals in each group. The criterion for counting an individual TH-positive neuron was the presence of its nucleus either within the counting frame, or touching the right or top frame lines, but not touching the left or bottom lines. Relative density of striatal TH-positive neuronal fibers and immunofluorescence intensity were analyzed by Image-Pro Plus 6.0 software.

### Determination of levels of dopamine and its metabolites, 3, 4-dihydroxyphenylacetic acid (DOPAC) and homovanilic acid (HVA)

The striatum of six to eight animals of each group was added with a certain volume of homogenate (0.1 M perchloric acid (HClO_4_) containing 0.01% EDTA) by weight (10 μL/mg), then centrifuged twice at 10,000 g at 4 ^o^C for 10 min to obtain supernatants. The analysis method was followed previously described [40], a high-pressure liquid chromatography (HPLC) system coupled to a 2465 electrochemical detector (Waters) were used to measure the concentrations of DA and its metabolites.

### Western blot assay

Western blot assay was executed as previously reported [35]. Briefly, striatum and substantia nigra tissues were collected and ultrasonic in indicated volumes of RIPA lysis buffer containing a cocktail of protease and phosphatase inhibitors (Santa Cruz, USA) in ice-cold water for 10 min. After 20 min centrifugation at 14,000 g at 4 ^o^C, the whole protein concentrations were determined by the BCA assay (Pierce, Rockford, IL, USA). SDS sample buffer was used to dilute the cell lysates and the protein (20-40 μg) was separated on a 12% SDS–polyacrylamide gel. After blocking with a blocking buffer, the polyvinyldifluoride membranes were co-incubated using primary antibodies overnight at 4 ^o^C respectively against TH (Cell Signaling Technology), CDK5, phospho-Ser444-MEF2D (p-MEF2D, Merck Millipore, Germany), MEF2D (Merck Millipore, Germany), PGC1α, Nrf2, HO-1, TFAM, phospho-Ser473-Akt (p-Akt), total-Akt (Akt), p-GSK3β, total-GSK3β (GSK3β), phospho-Ser133-CREB (p-CREB, Cell Signaling Technology), CREB (Cell Signaling Technology), mature BDNF (abcam, Britain), synaptophysin (SYP, Cell Signaling Technology) and β-actin. After incubation with the secondary antibodies at room temperature for 2 h, the signals were obtained using an ECL Plus kit (Fude Biological Technology Co. Ltd, China) and exposed to Kodak autoradiographic films. Finally, the quantitative analysis was provided by Carestream MI SE system.

### Primary cerebellar granule neuron cultures

Cerebellar granule neurons (CGNs) were obtained from 7-8 day old Sprague-Dawley rats (Laboratory Animal Center of Guangdong Province) as defined in our previous publication [34]. Cells were co-incubated at a density of 1.0-1.5×10^6^ cells/mL in basal modified Eagle’s (BME) medium supplemented with 10% fetal bovine serum, 25 mM KCl, 2 mM glutamine. After 24 h seeding, 10 μM cytosine arabinoside was supplemented to the culture medium to inhibit the growth of glial cells. CGNs cultured at 7 days *in vitro* (DIV) were used for the experiments.

### Differentiation protocol for dopaminergic neurons

Differentiation of human induced pluripotent stem cells (hiPSCs) into A9-type dopaminergic neurons (the type of neurons initially injured in PD) was performed as previously reported [57]. In brief, Accutase was used to dissociate the colonies into a single cell suspension during the early differentiation. Single cells were collected and re-placed on matrigel and gelatin (BD, Biosciences, USA) respectively, coated 6-well plates to purify hiPSCs and eliminate fibroblast feeders. After five stages of differentiation by changing different cultured media, the designated dopaminergic neurons were obtained and finally used in the following experiments.

### Neurotoxicity assay

CGNs were inoculated on 96-well plates and co-incubated with various concentrations of T-006 and TMP for 2 h followed by neurotoxins (MPP^+^ or 6-OHDA)-induced injury for 24 h. The cell viability was assessed by the 3-(4, 5-dimethylthiazol-2-yl)-2, 5-diphenyltetrazolium bromide (MTT) test. Mitochondrial membrane potential (Δψm) was measured with JC-1 (Beyotime Biotechnology, China) staining. Intracellular ROS (reactive oxygen species) was detected by H_2_DCF-DA probe (10 μM, Sigma Aldrich) and intracellular ATP levels were analyzed using the CellTiter-Glo luminescent cell viability assay kit (Promega). A minimum of four reduplicates were analyzed for each condition in three independent trials.

### Hoechst assay

CGNs were cultured on 24-well plates and co-incubated with the specified concentrations of 10 μM T-006 and 100 μM TMP for 2 h, then directly exposed to 150 μM MPP^+^ for 24 h. Then cells were fixed with 4% PFA in a cell incubator for 20 min. After three washes with PBS, cells were permeabilized by using 0.3% Triton X-100 (sigma) for 15 min, subsequently blocked by 3% BSA for 30 min at room temperature. After 10 min incubation with Hoechst, nuclei were visualized using a fluorescence microscope at 400× magnification.

### Luciferase reporter gene assays

Differentiated A9 DA neurons derived from A53T mutant hiPSCs of a PD patient (A53T DA neurons) and isogenic-corrected ones, in which a SNCA-A53T mutation was corrected [57], were transfected in 24-well plates with a MEF2, PGC1α or antioxidant response element (ARE) luciferase reporter, along with the Renilla luciferase control vector using Amaxa Nucleofector II followed by the manufacturer’s instructions. After 12 h transfection, the cells were treated with the indicated concentrations compounds for 24 h. After cells lysis, the supernatants were collected and analyzed by a Dual-Glo luciferase assay kit (Promega) following the manufacturer’s guidance. Firefly luciferase activity was normalized to Renilla luciferase activity. Three independent trials with six reduplicates were performed in the luciferase reporter assay of the above four kinds of genes.

### Cell viability assay

CGNs of 1.0-1.5×10^5^ were transfected with MEF2D siRNA (Santa Cruz, CA) and scramble siRNA (Santa Cruz, CA) using Lipofectamine 2000 (Invitrogen, Carlsbad, CA, USA) as followed to the manufacturer’s instructions. After transfected with indicated RNA for 48 h, CGNs were co-incubated with T-006 (10 μM) or placebo for 2 h, then damaged to MPP^+^ for 24 h, and finally carried out the MTT assay to measure cell viability.

### RNA extraction and the reverse transcription polymerase chain reaction

Real-time PCR (RT-PCR) was executed on the cDNA obtained from total RNA (4 μg) as previously described [39]. Concretely, TOPreal qPCR 2× PreMix with SYBR green (Takara, Japan) and a CFX real-time PCR detection system (Bio-Rad) were involved in the complement of RT-PCR. The primer sequences used were as follows: CDK5, GGCTTCATGATGTCCTGCATA (forward) and GACAGAATCCCAGGCCTTTC qqnCATCCGGCTCTGGGCTGTCA (reverse); PGC1α, CCTCCATGCCTGACGGCACC (forward) and GAGCTGAGTGTTGGCTGGCG (reverse); Nrf1, CGCAGTGACGTCCGCACAGA (forward) and AAGGTCCTCCCGCCCATGCT (reverse); Nrf2, GCTATTTTCCATTCCCGAGTTAC (forward) and ATTGCTGTCCATCTCTGTCAG (reverse); GAPDH, GGGGCTCTCTGCTCCTCCCTG (forward) and CGGCCAAATCCGTTCACACCG (reverse). The relative quantification was calculated using the 2^−ΔΔct^ method with GAPDH as the internal control.

### Data analyses

One-way ANOVA with Dunnett's test for comparisons multiple experimental statistical. All results were conducted using ImageJ software and Prism 7 program (GraphPad Software, Inc.). In all case, differences were considered statistical significant if P values < 0.05.
